# Cecal volvulus in rural Kenya: delayed presentation contributes to high mortality

**DOI:** 10.1186/s12893-021-01416-8

**Published:** 2021-12-19

**Authors:** Kimutai R. Sylvester, Philip B. Ooko, Michael M. Mwachiro, Robert K. Parker

**Affiliations:** 1 Department of Surgery, Tenwek Hospital, PO Box 39, Bomet, 20400 Kenya; 2 Department of Surgery, AIC Litein Hospital, Litein, Kenya; 3 Department of Surgery, Alpert Medical School of Brown University, Providence, RI USA

**Keywords:** Cecal volvulus, Intestinal obstruction, Surgery, Kenya

## Abstract

**Background:**

Cecal volvulus, which is a torsion involving the cecum, terminal ileum, and ascending colon around its own mesentery, results in a closed-loop obstruction. It is a rare reported cause of adult intestinal obstruction. This study aimed to review the clinical presentation, management, and outcomes at a rural, resource-limited referral center.

**Methods:**

We performed a retrospective review of all patients with a diagnosis of cecal volvulus between January 1st, 2009 and December 31st, 2019 at Tenwek Hospital in Bomet, Kenya. The outcome of survival was compared by the time to presentation. Mortality was also compared with prior reports of intestinal obstruction at our institution.

**Results:**

Thirteen patients were identified with a mean age of 52 years and a mean symptom duration of 5 days. All patients presented with abdominal pain and distension. Seven patients (54%) presented with perforation, gangrene, or gross peritoneal contamination. Identified risk factors were Ladds bands with malrotation, adhesions, and a sigmoid tumor. Procedures included primary resection and anastomosis (7), damage control (3) with anastomosis on second-look in 2 of these, simple surgical detorsion (1), and surgical detorsion and cecopexy (2). There were four mortalities (31%), of which all had delayed presentation with perforation and fecal contamination. Delays to presentation were associated with mortality (p = 0.03). Cecal volvulus resulted in increased perioperative mortality compared to all intestinal obstructions presenting to the institution (p < 0.0001).

**Conclusions:**

Cecal volvulus carries a high risk of mortality. A high index of suspicion and early consideration in the differential diagnosis of intestinal obstruction should be considered to reduce the mortality associated with the delay in preoperative diagnosis.

## Introduction

Cecal volvulus refers to torsion involving the cecum, terminal ileum, and ascending colon around its own mesentery resulting in a closed-loop obstruction [1]. Cecal volvulus is the second most common form of colonic volvulus after sigmoid volvulus and represents approximately 1–1.5% of all adult intestinal obstruction [1]. Various reports have described cecal volvulus within the literature, typically with individual or few patients.

There is a paucity of data from the African continent of this condition, limited predominately to case reports [2, 3]. Despite reports of the relatively higher prevalence of sigmoid volvulus in East Africa [4, 5], there is limited data concerning cecal volvulus. A recent report described the experience with intestinal volvulus in Malawi. The investigators described 8 cases (1.6% of all patients with intestinal volvulus) of cecal volvulus but did not examine the impact of cecal volvulus on mortality [6]. We hypothesized that cecal volvulus carries a higher risk of mortality in rural Africa than other forms of intestinal volvulus or obstruction. This study aimed to review clinical presentation, management, and outcome of patients with cecal volvulus at a referral hospital in rural Kenya.

## Methods

We conducted a retrospective review of all adult patients’ paper and electronic records and included all with a diagnosis of cecal volvulus between January 1st, 2009 and December 31st, 2019 at Tenwek Hospital, a teaching and referral mission hospital in rural southwestern Kenya. Pediatric patients were excluded from review. An electronic medical record system was instituted mid-year, 2012. Demographic data, presenting symptoms and clinical features, operative findings including whether perforation was present as documented by the operating surgeon, procedures, and patient outcomes were reviewed.

Data were extracted from the medical record and stored in Microsoft Excel and analyzed in Stata version 16. Students t-test of the normally distributed data was performed to compare the duration of illness, in days, prior to presentation for the dichotomous patient outcomes of alive compared to dead at the time of hospital discharge. Comparison of perioperative mortality rates was conducted, using Fisher’s exact test, with other types of intestinal obstruction previously reported at our institution between November 2009 and October 2013 as described [7, 8].

Strengthening the reporting of observational studies in epidemiology guidelines were followed for the methods of this observational study. Informed consent was waived and ethics approval for this retrospective review was obtained from the Tenwek Hospital Institutional Ethics Review Committee.

## Results

Thirteen patients were identified with a diagnosis of cecal volvulus during the 11-year review period. Age at the time of diagnosis was a mean of 51 years (SD 23 years) and a median of 50 with a range of 25 to 84 years. Demographics are listed in Table 1. Eight (62%) were females, all younger than 65 years. Except for one (28 years), four men were over the age of 65 years.

**Table 1:** Demographics

Age (years)	Male (n = 5)	Female (n = 8)	Total (n = 13)
25–35	1 (20%)	4 (50%)	5 (38﻿%)
36–45	0	1 (13%)	1 (8%)
46–55	0	2 (25%)	2 (15%)
56–65	0	1 (13%)	1 (8%)
66–75	1 (20%)	0	1 (8%)
> 76	3 (60%)	0	3 (23%)

All patients presented with abdominal pain and distension, with peritonitis reported in 5 (38%) cases (Table 2).

**Table 2:** Symptoms and signs consistent with acute cecal volvulus

Symptom/Sign	Acute cecal volvulus (n = 13)
Abdominal distension	13 (100%)
Abdominal pain	13 (100%)
Constipation	12 (92%)
Vomiting	11 (85%)
Abdominal tenderness	11 (85%)
Nausea	8 (62%)
Peritonitis	5 (38%)
Empty rectum on digital rectal exam	3 (23%)
Bloody mucoid discharge	1 (8%)

**Fig. 1:**
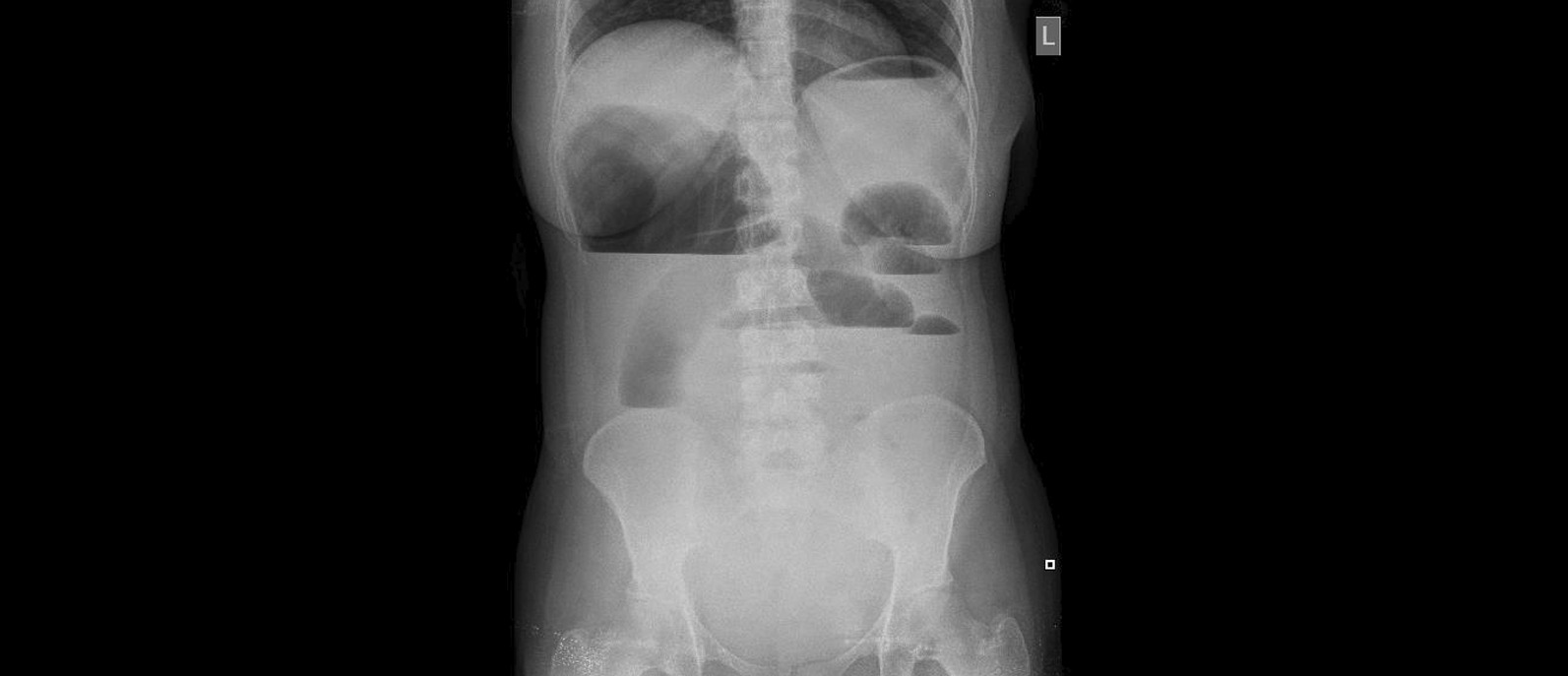
Erect abdominal radiograph showing dilated small bowel air-fluid levels with distended colon in right-upper quadrant

**Fig. 2:**
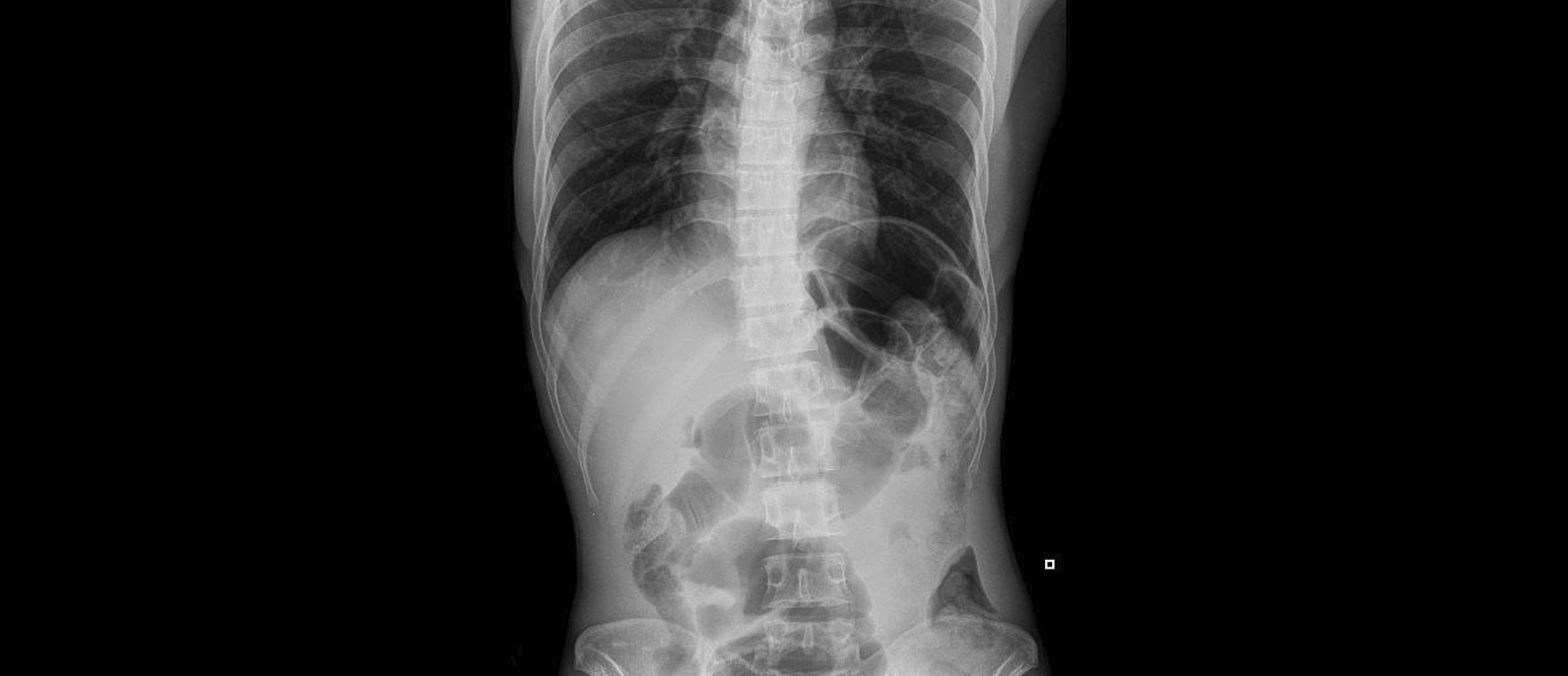
Plain erect abdominal radiograph showing distension of colon in the right lower quadrant-pelvis region

The mean and median symptom duration was 5 days (Table 3). The only imaging modality used in our series was plain abdominal radiography with examples in Figs. 1 and 2. Leukocytosis and hypotension were present in most, but not all of the patients with perforation, gangrene or gross peritoneal contamination. All patients received perioperative antibiotic prophylaxis. All six patients with viable cecum did not have leukocytosis or hypotension at presentation. Perforation occurred in 6 patients, 4 of those died (67%). Among patients who died, they presented with greater delays (average 6.5 days, standard deviation 1.7) than those who were alive at follow-up (average 3.8 days, standard deviation 1.8) (p = 0.03). One patient developed fascial dehiscence with subsequent bowel evisceration. The mean hospital duration was 6 days.

**Table 3:** Summary of all patients’ characteristics

Age	Sex	Time to presentation (days)	Hypotension	Leukocytosis	Hospital stay (days)	Cecal status	Outcome (alive/dead)	Operation
65	F	3	No	No	6	Viable	Alive	Right hemicolectomy
27	F	2	No	No	5	Viable	Alive	Detorsion and cecopexy
25	F	5	Yes	Yes	6	Gangrene	Alive	Right hemicolectomy
50	F	7	No	No	7	Perforated	Alive	Right hemicolectomy
70	M	7	Yes	Yes	5	Perforated	Dead	Right hemicolectomy; 2nd look anastomosis
79	M	5	No	No	6	Viable	Alive	Right hemicolectomy
31	F	1	Yes	No	7	Perforated	Alive	Right hemicolectomy
53	F	5	No	No	7	Viable	Alive	Right hemicolectomy
83	M	8	No	Yes	6	Perforated	Dead	Right hemicolectomy
45	F	4	Yes	Yes	11	Perforated	Dead	Right hemicolectomy; 2nd look anastomosis
84	M	7	No	No	1	Perforated	Dead	Damage control surgery
28	M	3	No	No	3	Viable	Alive	Simple detorsion
29	F	3	No	No	5	Viable	Alive	Detorsion and cecopexy

Contributing risk factors were established intra-operatively in four cases: Ladds bands with malrotation (n = 1), adhesions (n = 2), and a sigmoid tumor (n = 1). Procedures performed included primary resection and anastomosis in 7 (54%), damage control surgery with resection and anastomosis on second look in 2 (15%), damage control surgery only in 1 (8%), simple surgical detorsion in 1 (8%), and surgical detorsion and cecopexy in 2 (15%).

Perioperative mortality was increased among patients with cecal volvulus (31%) when compared to a prior report of all intestinal obstructions requiring operative intervention at our hospital (5%) (p < 0.0001) [7].

## Discussion

Numerous patients with cecal volvulus presented to Tenwek Hospital during the study period. Delays in diagnosis and advanced disease resulting in necrosis and perforation were frequent. This series describes a high mortality rate (31%), especially among patients with delayed presentation to the hospital. Prior reports have described higher mortality rates when gangrene is present [9, 10]. The emergent nature of the operations in this series likely increases the mortality when compared to other reports [11, 12] as all patients who experienced mortality were perforated at the time of operation. Compared to other causes of intestinal obstruction and volvulus at our institution, cecal volvulus carries a higher risk of mortality. To improve patient outcomes, careful attention must be paid to the prompt recognition of cecal volvulus to decrease the delays to presentation.

Patients with cecal volvulus may present with highly variable clinical signs and symptoms, thus posing a diagnostic challenge [13]. Factors that have been implicated in the causation of cecal volvulus include prior abdominal surgeries [14], late-term pregnancy [15], high-fiber diet, adynamic ileus, chronic constipation, and distant colon obstruction [1]. Intra-operative findings support the prerequisite factor of anatomical predisposition including a mobile cecum [2], with some authors citing cultural and dietary influences on intestinal motility [1, 16]. Case reports have noted unique circumstances associated with cecal volvulus occurring through the foramen of Winslow [17], precipitated by acute appendicitis [18], following colonoscopy [19], immediately after cardiac surgery [20], or the presence of a Meckel’s diverticulum [21] or uterine leiomyoma [22]. Depending on the presence of colon viability and intestinal gangrene, mortality of various series ranges from 10 to 40% [13, 23, 24], which is consistent with our experience.

The majority of our patients were referred from other peripheral facilities unable to provide surgery. The clinical and symptom pattern of patients with necrotic cecum was similar to patients with viable intestine [25]. Imaging techniques commonly employed in the diagnosis of cecal volvulus include plain abdominal radiography, barium enema, abdominal computed tomography scan and colonoscopy; however, only plain abdominal radiography was utilized in this study. Their sensitivity increases in the presence of typical symptoms and signs [13]. Up to 30% of patients do not show radiographic peculiarities, making diagnosis difficult with consequent delay [13]. Plain abdominal radiography is the most readily available form of imaging in many resource-limited settings in patients with a clinical presentation of acute intestinal obstruction. Acute volvulus presents with cecal dilatation, air-fluid levels, proximal small bowel dilatation, and absence of gas in the distal colon or even presence of pneumo-peritoneum in cases of perforated bowel. Radiography lacks specificity, only detects half of cases [24, 26–28], and is therefore insufficient to confirm cecal volvulus [24]. In contrast to plain abdominal radiography, barium enema is diagnostic in more than 90% of cases [1, 26, 28, 29]. Typical patients present with a ‘beaked’ termination of the column of contrast with a lack of filling of the cecum [1, 23]. However, it should not be used for patients who present with signs of ischemia such as hypotension and peritonitis suggestive of sepsis secondary to gangrenous or perforated bowel. Computed tomography, with ‘coffee bean’, ‘bird beak, and ‘whirl’ signs, typically reveals the presence and location of the volvulus and allows the early identification of ischemia and perforation [24, 29, 30]. Computed tomography became more readily available at the end of the study period, and could improve accurate pre-operative diagnosis.

**Fig. 3:**
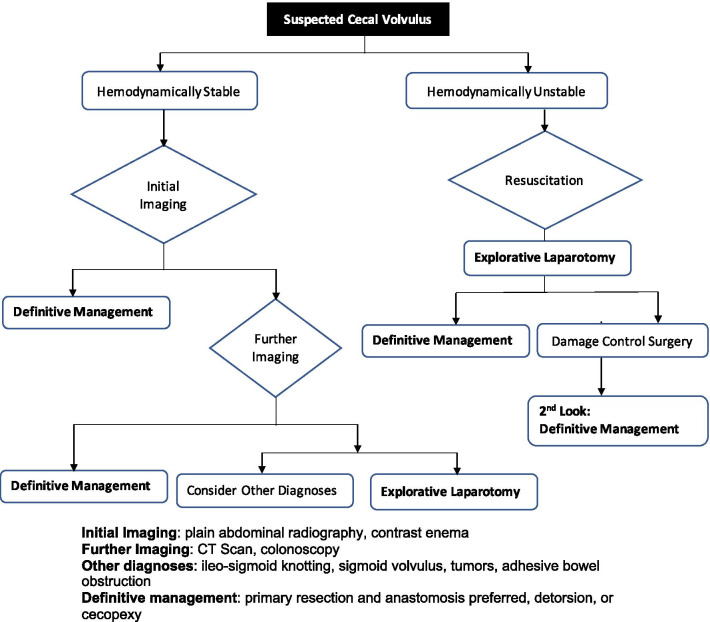
Management protocol for suspected cecal volvulus

All identified patients received operative management. Primary resection (right hemicolectomy) and anastomosis (end to side ileo-transverse) was preferred in patients with viable bowel as it has been associated with low recurrence rates [11]. Several options can be available in patients with a viable colon such as detorsion or cecopexy with tube cecostomy being mostly abandoned due to associated complications [1, 10, 11, 16]. Non-operative decompression is rarely achievable [10]. Therapeutic barium enema has a low success rate [1], and is therefore not recommended. Some series report about 30% success rate for colonoscopy [1] with the recurrence rate exceeding 50% [30]. Cecopexy is frequently advocated in older and debilitated patients when the bowel is viable [11, 31] as it has been described to have low mortality, morbidity and recurrence rates [10, 27, 28]. The growing use of minimally invasive techniques has seen the role of cecopexy, colopexy and cecostomy increasing [10, 32], especially in elective cases [33]. However, in the presence of gangrene or perforation of the cecum, the definitive management would be resection and primary end-to-side or side-to-side ileocolic anastomosis or resection with ileostomy. Damage control surgery, which involves resection of gangrenous or perforated segment and temporarily leaving the bowel in discontinuity, was preferred in hemodynamically unstable patients to allow for resuscitation before the second look operation for definitive surgery [11]. Due to the venous outflow obstruction of intestinal volvulus, this approach allows the bowel to demarcate for definitive resection at the second operation. This approach has been facilitated by the growing ability to care for critically-ill patients and the establishment of critical care units in our hospital [34]. However, many patients still die of multi-organ failure secondary to refractory septic shock. Mortalities occurred after delayed presentation with perforation, likely due to limited available resources and inadequate expertise to recognize and initiate prompt treatment in the surrounding peripheral hospitals. One patient, in our series, who underwent surgical detorsion and cecopexy had an acute on chronic presentation with scarred mesentery. Although we have no evidence of recurrence, follow-up for most patients in the study population, whether medical or surgical, is challenging as patients often do not present to the hospital unless there is a problem. We outline our proposed management strategy in Fig. 3.

A fascinating theory by Bauman et al. proposed an association between long-distance running and cecal volvulus [35]. Although we did not review this in our patients’ history, the surrounding area of rural Kenya in which Tenwek Hospital is located is renowned for producing marathon champions; thus, raising an interesting avenue for future investigation. Cecal volvulus is a rare diagnosis worldwide with numerous case reports and case series. It seems to be more common in our setting than in other reported populations though this cannot be determined by our retrospective experience.

Our study is limited by the relatively small sample size; however, given the infrequent diagnosis of cecal volvulus, most reports have even fewer patients available for review. This limited number of outcomes precludes further statistical analysis to control for potential confounding factors. Although we were unable to collect the time from presentation to operation due to the retrospective nature of the review, we believe that the delays in units of days prior to presentation far outweigh the minutes to possibly hours upon arrival to the hospital. It is possible that cases that did not undergo laparotomy to establish the diagnosis were missed by clinicians. Future studies should investigate the diagnosis of cecal volvulus throughout multiple centers in the region.

In conclusion, the etiology of cecal volvulus is multifactorial, with an underlying anatomic predisposition. In a region known to have a high incidence of sigmoid volvulus, there may also be higher rates of cecal volvulus. Prompt diagnosis, with awareness of the potential of cecal volvulus, is required to avoid life-threatening complication of bowel ischaemia, necrosis, and perforation. Surgery is both diagnostic and therapeutic in the absence of a conclusive preoperative diagnosis and access to surgical care is paramount. Resection and anastomosis is the preferred method of definitive treatment depending on the general condition of the patient. This series reports a very high mortality rate associated with the condition. With improving healthcare facilities and capabilities, prompt recognition and treatment will hopefully improve outcomes.

## Data Availability

All data generated or analyzed during this study are included in this published article.

## References

[1] Consorti E, Liu T (2005). Diagnosis and treatment of caecal volvulus. Postgrad Med J.

[2] Mwita C, Muthoka J, Kanina P, Mulingwa P (2014). Caecal volvulus in an adolescent African male: a case report and brief review of the literature. Pan Afr Med J.

[3] Mazine K, Elbouhaddouti H, Toughrai I, Mouaqit O, Benjelloun E, Ousadden A, Taleb KA (2017). Volvulus of the cecum: a rare cause of intestinal occlusion: about two cases. Pan Afr Med J.

[4] Chalya PL, Mabula JB (2015). Sigmoid volvulus and ileo-sigmoid knotting: a five-year experience at a tertiary care hospital in Tanzania. World Journal of Emergency Surgery.

[5] Mulugeta GA, Awlachew S (2019). Retrospective study on pattern and outcome of management of sigmoid volvulus at district hospital in Ethiopia. BMC Surg.

[6] Purcell LN, Reiss R, Mabedi C, Gallaher J, Maine R, Charles A (2020). Characteristics of intestinal volvulus and risk of mortality in Malawi. World J Surg.

[7] Ooko PB, Sirera B, Saruni S, Topazian HM, White R (2015). Pattern of adult intestinal obstruction at Tenwek hospital, in south-western Kenya. Pan Afr Med J.

[8] Ooko PB, Saruni S, Oloo M, Topazian HM, White R (2016). Ileo-sigmoid knotting: a review of 61 cases in Kenya. Pan Afr Med J..

[9] O'Mara CS, Wilson TH, Stonesifer GL, Cameron JL (1979). Cecal volvulus: analysis of 50 patients with long-term follow-up. Ann Surg.

[10] Madiba T, Thomson S, Church JM (2002). The management of cecal volvulus. Dis Colon Rectum.

[11] Martin M, Steele S (2010). Twists and turns: a practical approach to volvulus and instussusception. Scand J Surg.

[12] Grossmann E, Johnson F, Enger K, Leake B, Virgo K, Longo W (1999). Cecal volvulus: outcome of management by celiotomy. Tech Coloproctol.

[13] Pulvirenti E, Palmieri L, Toro A, Di Carlo I (2010). Is laparotomy the unavoidable step to diagnose caecal volvulus?. Ann R Coll Surg Engl.

[14] Ferguson L, Higgs Z, Brown S, McCarter D, McKay C (2008). Intestinal volvulus following laparoscopic surgery: a literature review and case report. J Laparoendosc Adv Surg Tech.

[15] Singla SL, Kadian YS, Goyal A, Sharma U, Kadian N (2005). Caecal volvulus in pregnancy: is delay in diagnosis avoidable?. Asian J Surg.

[16] Gingold D, Murrell Z (2012). Management of colonic volvulus. Clin Colon Rectal Surg.

[17] Kamyab A (2016). Cecal herniation and volvulus through the foramen of winslow. Am Surg.

[18] Bhatti S, Khan MA, Farooka W, Butt UI, Rehman UA, Malik AA (2017). An unusual case of caecal volvulus due to appendicitis, successfully managed by caecopexy. J Coll Physicians Surg Pak.

[19] Beltzer C, Geiger A, Schmidt R, Danz B, Maier A, Karpa R, Dikopoulos N (2017). A rare case of coecal volvulus after colonoskopy due to a mobile coekum-diagnosis, surgical therapy and postoperative complications. Z Gastroenterol.

[20] Muller D (2014). Caecal volvulus after cardiac surgery. Case Rep.

[21] Altaf A, Aref H (2014). A case report: Cecal volvulus caused by Meckel's diverticulum. Int J Surg Case Rep.

[22] de Vries HS, Samlal RK, Maresch BJ, Hoven-Gondrie ML (2015). Cecal volvulus caused by a large uterine leiomyoma. Int J Surg Case Rep.

[23] Katoh T, Shigemori T, Fukaya R, Suzuki H (2009). Cecal volvulus: report of a case and review of Japanese literature. World J Gastroenterol: WJG.

[24] Swenson BR, Kwaan MR, Burkart NE, Wang Y, Madoff RD, Rothenberger DA, Melton GB (2012). Colonic volvulus: presentation and management in metropolitan Minnesota, United States. Dis Colon Rectum.

[25] Güller U, Zuber M, Harder F (2001). Cecum volvulus–a frequently misdiagnosed disease picture. Results of a retrospective study of 26 patients and review of the literature. Swiss Surg.

[26] Folaranmi SE, Cho A, Tareen F, Morabito A, Rakoczy G, Cserni T (2012). Proximal large bowel volvulus in children: 6 new cases and review of the literature. J Pediatr Surg.

[27] Gupta S, Gupta S (1993). Acute caecal volvulus: report of 22 cases and review of literature. Ital J Gastroenterol.

[28] Wright TP, Max M (1988). Cecal volvulus: review of 12 cases. South Med J.

[29] Ruiz-Tovar J, García PC, Castiñeiras VM, Molina EM (2009). Caecal volvulus: presentation of 18 cases and review of literature. Cirugía Española (English Edition).

[30] Moore CJ, Corl FM, Fishman EK (2001). CT of cecal volvulus: unraveling the image. Am J Roentgenol.

[31] Tejler G, Jiborn H (1988). Volvulus of the cecum. Dis Colon Rectum.

[32] Gomes CA, Soares C, Catena F, di Saverio S, Sartelli M, Gomes CC, Gomes FC (2016). Laparoscopic management of mobile cecum. J Soc Laparoendosc Surg..

[33] Sakamoto Y, Hiyoshi Y, Sakata K, Toyama E, Takata N, Yoshinaka I, Harada K, Baba H (2018). Case of cecal volvulus successfully treated with endoscopic colopexy. Asian J Endosc Surg.

[34] Many HR, Otoki K, Parker AS, Parker RK (2021). Implementation of a surgical critical care service reduces failure to rescue in emergency gastrointestinal surgery in rural Kenya. Ann Surg.

[35] Bauman BD, Witt JE, Vakayil V, Anwer S, Irwin ED, Kwaan MR, Pruett TL, Harmon JV (2019). Cecal volvulus in long-distance runners: a proposed mechanism. Am J Emerg Med.

